# Core information sets for informed consent to surgical interventions: baseline information of importance to patients and clinicians

**DOI:** 10.1186/s12910-017-0188-7

**Published:** 2017-04-26

**Authors:** Barry G. Main, Angus G. K. McNair, Richard Huxtable, Jenny L. Donovan, Steven J. Thomas, Paul Kinnersley, Jane M. Blazeby

**Affiliations:** 1 Centre for Surgical Research, School of Social and Community Medicine, University of Bristol, Canynge Hall, 39 Whatley Road, Bristol, BS8 2PS UK; 2 School of Oral and Dental Sciences, University of Bristol, Bristol, BS1 2LY UK; 3 Division of Surgery, Head and Neck, University Hospitals Bristol NHS Foundation Trust, Bristol, BS2 8HW UK; 4 Centre for Medical Education, University of Cardiff, Cardiff, CF14 4YU UK

**Keywords:** Informed consent, Information, Shared-decision making, Autonomy

## Abstract

**Background:**

Consent remains a crucial, yet challenging, cornerstone of clinical practice. The ethical, legal and professional understandings of this construct have evolved away from a doctor-centred act to a patient-centred process that encompasses the patient’s values, beliefs and goals. This alignment of consent with the philosophy of shared decision-making was affirmed in a recent high-profile Supreme Court ruling in England. The communication of information is central to this model of health care delivery but it can be difficult for doctors to gauge the information needs of the individual patient. The aim of this paper is to describe ‘core information sets’ which are defined as a minimum set of consensus-derived information about a given procedure to be discussed with all patients. Importantly, they are intended to catalyse discussion of subjective importance to individuals.

**Main body:**

The model described in this paper applies health services research and Delphi consensus-building methods to an idea orginally proposed 30 years ago. The hypothesis is that, first, large amounts of potentially-important information are distilled down to discrete information domains. These are then, secondly, rated by key stakeholders in multiple iterations, so that core information of agreed importance can be defined. We argue that this scientific approach is key to identifying information important to all stakeholders, which may otherwise be communicated poorly or omitted from discussions entirely. Our methods apply systematic review, qualitative, survey and consensus-building techniques to define this ‘core information’. We propose that such information addresses the ‘*reasonable* patient’ standard for information disclosure but, more importantly, can serve as a spring board for high-value discussion of importance to the *individual* patient.

**Conclusion:**

The application of established research methods can define information of core importance to informed consent. Further work will establish how best to incorporate this model in routine practice.

## Background

Knowing what quantity and quality of information best prepares patients to authorise medical procedures is an enduring problem facing doctors. This issue has come to prominence again with the recent English Supreme Court ruling in *Montgomery* [1]. Now, the law is aligned more closely with professional standards that require clinicians to spend time with patients discussing the risks, intended benefits, and reasonable alternative options before seeking consent to proceed. The idea is that a process of shared decision-making takes place that results in the doctor and patient agreeing on an appropriate treatment plan that best fits the patient’s goals, circumstance and beliefs. The era of the ‘reasonable doctor’ standard for information disclosure has passed. The clinician is responsible for finding out what risks or complications associated with the intervention are material to a reasonable person in the patient’s position or would probably be material to the individual patient consenting to that procedure [1].

But this is difficult. How does the clinician begin to explore what information should be disclosed to any given patient? How does the patient, often with little or no baseline knowledge or understanding of what is being proposed, start to formulate ideas about materiality and weigh-up treatment options, so as to be ultimately satisfied with decisions made? It is apparent that the issue is more complex than simply quantifying an amount of information to be disclosed. The means of communicating that information to ensure that it is useful and understandable to the patient must be considered. It is this framing of consent as a function of patient-centred communication and shared decision-making that appears to be the major shift that has come with *Montgomery*.

This paper tracks the evolution of the patient-centred narrative in the law around informed consent to medical intervention. The emphasis is on how information disclosure might facilitate autonomous authorisation. Pertinent bioethical theory is discussed before describing the rationale and methods for a model that aims to provide a baseline from which informed consent consultations involving discussions about invasive and/or high-risk interventions might better meet the needs of patients and clinicians alike. The focus is on how the model fits alongside current English law but there is relevance to other jurisdictions with similar informed consent standards.

### From *Bolam*, *Sidaway*, and *Pearce* to *Montgomery*

Testing whether or not information disclosed by doctors has been sufficient to permit informed, autonomous decision-making by patients has dominated case law around informed consent over recent decades. In the UK, several landmark cases serve to track the evolution from physician-centred to patient-centred expectations for standards of care. The ‘prudent doctor’ standard for information disclosure, established in the wake of the *Bolam* case in 1957 (Mr Bolam sustained limb fractures during electroconvulsive therapy) stated that a doctor would not be found liable in negligence if he or she acted in accordance with a practice accepted as proper by a responsible body of medical professionals skilled in that particular art [2]. Thus viewed, information disclosure was regarded as a skill possessed by medical professionals, alongside their diagnostic and therapeutic abilities, and for which the standards of practice were determined by the profession. It was not until *Sidaway* some 30 years later that the English courts began to acknowledge the paternalistic nature of the *Bolam* test [3]. The claimant was unsuccessful in her claim for damages resulting from not being properly informed about the risks of spinal surgery, but the dissenting opinion of one of the Law Lords - that patients should ordinarily be warned of material risks, and thus rejecting the *Bolam* test - was notable. Further modifications, and recognition that materiality should be about patients and not doctors, came in *Pearce* (an obstetric case in which the claimant alleged being insufficiently informed about the risks of delaying labour) and *Chester* (in which a neurosurgeon was found negligent in his duty to inform about the risks of a spinal operation) [4, 5]. But it was not until the 2015 ruling in *Montgomery*, when it was ruled that a pregnant diabetic woman should have been informed about the risk of a birth complication that subsequently arose, that there was an emphasis in English law on the need to find out what is material to the individual patient: ‘[a risk is also material] if the doctor is, or should reasonably be, aware that the particular patient would be likely to attach significance to it’ [6].

The Supreme Court judges affirmed that it is for the patient, and not the doctor or medical establishment, to decide upon the materiality of risk [6–8]. The *Bolam* test is no longer relevant. Clinicians are required to engage with patients in conversations that reveal these important issues of materiality, treatment preferences, and expected outcomes. While this appears to be a welcome shift, questions remain about whether or not patients will be more satisfied with, and have a better understanding of, the information provided and discussed in the consent consultation. One criticism of the approach advocated in *Montgomery* might be that it is an abstract, cryptic construct that only serves to further confuse clinicians and might encourage disclosure of very large amounts of information [7]. Truly person-centred, or subjective, information disclosure is a laudable aim but it is not yet known whether it can be a realistic one [9].

Patient-centred care is now a major principle for healthcare delivery, and models like ‘shared decision-making’ require a collaborative deliberation between the patient and doctor that results in a mutually satisfactory treatment choice [10]. The ruling in *Montgomery* aligns the constructs of informed consent and shared decision-making more closely, and emphasises the need for a process rather than a single act [8]. The common thread running through these constructs is information: information that is disclosed, exchanged, useful, and understandable. The nature of this information is likely to be different for each patient but we hypothesise that there is a common, core amount of information that is not only important to all patients (‘prudent’ patients) but is also required to catalyse those discussions that meet the subjective information needs of individuals. This model offers clinicians a framework on which to base consent consultations that better meet the requirements laid down in *Montgomery*. Before describing our methodological approach to addressing our hypothesis, the next section outlines the rationale for ‘core information sets’ for informed consent.

### Core information sets

Our model is built around an interpretation of the ‘core disclosure’ concept described in Faden and Beauchamp’s seminal text [11]. The underlying premise is that while truly patient-centred, subjective information disclosure and exchange may be desirable, most patients require some level of baseline information on which to base further discussion, formulate questions, and make decisions about their health. Healthcare information may now be more readily available but it is increasingly complex. In addition, there can be concerns over its accuracy and relevance, and there may be no evidence that the patient understands it. Furthermore, variation in the quality and quantity of information disclosed in consent consultations was highlighted as a problem in *Montgomery*. It appears apt, therefore, to address this issue with the aim of improving practice. The ‘core disclosure’ approach - whereby a baseline amount of information is identified as important by key stakeholders – is, therefore, as relevant now as it was 30 years ago. It has the potential to catalyse information exchange that is of value to the patient, but crucially also recognises the clinician’s role in informed consent consultations. As far as we are aware, our methodological approach to developing this idea is novel. We have approached it pragmatically by applying scientific methods. Before outlining these, the intended scope and use of core information sets will be described, in order to contrast them with existing models for consultations, namely shared decision-making using decision aids.

### Defining the scope of the core information set

A core information set is intended for use when a patient is ready for a detailed discussion about a treatment recommendation or decision. To date, we have developed core information sets in three areas of surgical oncology where an operation is a major component of a patient’s treatment. It is envisaged that core information sets will form the basis of encounter tools [12] that help guide the patient, their families, and clinicians through the information domains that have been derived through consensus-building work. Although conceptually similar to patient decision aids [10], a single core information set-as presently conceived-does not directly compare the outcomes associated with different treatment options. Rather, core information sets are intended to ensure that information that has been shown to be important to patients, but often not discussed by doctors, is included in pre-operative consultations.

Defining the procedure to which the core information set applies is, therefore, required at the outset. It is important to clarify at this point that CIS are not intended as a panacea for information provision. We do not advocate a ‘tick box’ approach to informed consent consultations but instead aim to provide baseline information that is of proven importance to patients and clinicians, and which acts as a springboard for further discussion. Information about viable alternative therapeutic approaches must be included in prior discussions. The core information set is, however, intended as a focussed starting point for detailed conversation about a particular procedure once that procedure has been agreed upon, meaning that alternative procedures will need a similar disclosure of information. The potential strengths and weaknesses of this approach are explored later, but first we outline the methods we have used to define core information sets for securing the informed consent of patients to particular surgical procedures.

### Methodological approach

**Fig. 1:**
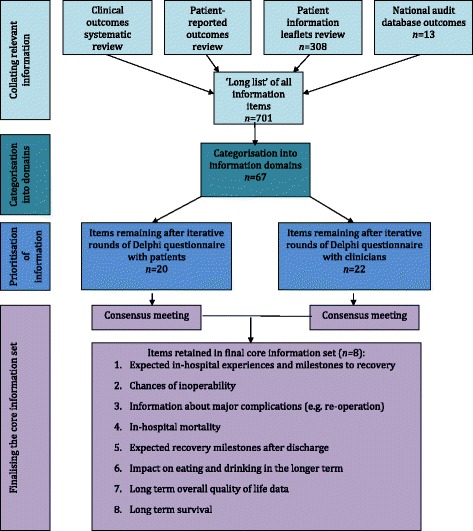
Phases in the development of a core information set for consent to oesophagectomy [13]

Our mixed-methods approach applies health services research and consensus-building techniques. Initially, a wide net is cast that aims to capture information that may be important for informed consent. Interviews and surveys using scientific methods, and involving key stakeholders, distill this large amount of information down to a core set. The overall methodological approach is outlined in the next four sub-sections. A more detailed description of the application of the methods have been published elsewhere [13], so the emphasis here is on explaining how a pragmatic scientific approach can be applied to our theoretical model for informed consent. Development of core information sets involves four main phases, as shown in Fig. 1. As will be seen, some of this work involves in-depth, iterative qualitative inquiry that is resource intensive to do well. Where possible, therefore, we have identified alternative methods that may better suit researchers with different expertise and/or resources.

### Collating relevant information

Information that may be relevant for discussion during the informed consent process can be collated from several sources (Table 1). Sensitive, systematic literature searches can identify papers reporting those clinical and patient-reported outcomes that might inform decision-making or treatment guidelines. Included articles should ideally reflect current practice, and consideration ought to be given to the time frame for the measurement of outcomes (e.g. short versus long term). Those outcomes of interest can be recorded verbatim from the papers for later consideration.

**Table 1:** Potential methods for gathering information of importance to clinicians and patients

• Written information
◦ Scientific papers reporting clinical and/or patient-reported outcomes of relevance
◦ Written information leaflets provided for patients by hospitals and other organisations
◦ Websites produced by charities, hospital units, and patient support groups
◦ National audit, guidelines, and policy documents
• Information of importance to individuals
◦ Delphi questionnaires
◦ Nominal group techniques
◦ Focus groups
◦ Individual in-depth interviews with clinicians and patients
◦ Recordings of consultations
◦ Individual questionnaires
◦ Patient experience resources, including websites (e.g. HealthTalk On Line)

Information included in patient information leaflets (PILs) provided by hospitals, specialty associations, and national charities can be collated. This is important because patients and their families are known to use these resources to augment information discussed in clinic [14]. These PILs contain information about, for example, the process of care and surgery that may be relevant for informed consent but not communicated in the scientific literature described above. It is important, however, to take account of potential inaccuracies contained within them [15]. Rather than relying on PILs as reliable sources of, for example, complication rates, it is recommended that the categories of information are recorded for further consideration later in the process. Additional sources of written healthcare information that may be analysed include national guidelines and audit documents. There is likely to be a large amount of potentially relevant information on internet web pages that could be included here.

Patients and clinicians can be given opportunities to report information of particular importance to them, but which may not be captured by other methods. Qualitative research methods such as non-participant observation and semi-structured interviews, for example, allow in-depth exploration of what information is exchanged during the consent process, and what information is most valued by patients [16]. Audio recordings of consent consultations will identify what information is discussed during consultations, and how questions are asked by patients and answered by clinicians. More simple methods for allowing patients and clinicians to express their views about what information is important include open-ended questions incorporated into surveys or questionnaires used in other stages of CIS development.

Careful consideration should be given to which key stakeholder groups are included in this phase of work. Different groups are likely to place different emphases on different types of information, and it is essential that patients are consulted. Healthcare workers may be more homogenous in their views compared with groups of patients, particularly when considering information about quality of life, body image, or social functioning [17]. It is important that sufficient patient representation is included to allow these differing views to be captured. We have found that purposively including patients at varying stages along the pre- and post-treatment trajectory leads to a comprehensive capture of important issues about which patients would value information. Expert patients, patient support group members, and family members of patients have all participated in our work to add depth to the views captured during the consensus-building phases.

At the end of this phase, additional information items identified are added to the ‘long list’ that is collated following reviews of the scientific and other written information.

### Categorising the information

There will be significant duplication and overlap in the information items collated in the initial phase of work. Duplicated items can be combined into broader ‘domains’ of healthcare information [13]. Thirty- and 90-day mortality would, for example, be included in a ‘mortality’ domain. This is a key step in the development of CIS because inappropriate categorisation of information may allow important meanings or differences to be lost. Both patients and clinicians can be involved in a ‘think aloud’ approach, whereby the participant is guided through the process by a researcher and asked to place each item of information in an appropriate domain that includes similar items [18]. Decisions are documented for transparency and subsequent peer review. The final domain list is then taken forward for prioritisation by stakeholders.

### Prioritising the information

Consensus-building methods can be applied to allow key stakeholders, including patients, to prioritise the information domains. The Delphi process, for example, is a survey technique that aims to guide independent, anonymous participants towards consensus in a structured way [19]. A principal advantage is that it can be conducted by post or email in a participant’s own time and space, meaning the potential influence of dominant personalities potentially encountered in a face-to-face group setting is minimised [20].

There is no statistical way of determining sample size for Delphi studies. We recommend selecting a sample size based on numbers of stakeholder groups, and anticipated variation based on key characteristics. As described above, it may be reasonable to expect healthcare professionals to be more homogenous in their views than patients. This technique is, however, time consuming and can involve a significant administrative burden. There is the potential, as with other questionnaire techniques, of significant attrition or failure-to-respond. It is important, therefore, to pilot any questionnaire with representatives of stakeholder groups to ensure the questionnaires are user friendly, with easy-to-follow instructions and straightforward layout, to help maximise fidelity. We have found patient support group representatives to be extremely valuable in this process. Depending on resources and expertise, therefore, a focus group or other method of building consensus may be more appropriate [19].

If a Delphi-approach is taken to elicit expert opinion, the information domains formulated in the previous phase are formatted into a questionnaire. Participants are asked to prioritise the list of domains on a Likert scale ranging from “not important” to “extremely important”. Cut-off criteria are applied to determine which items should be retained and which should be discarded. There is no agreement about how best to determine these criteria in Delphi studies [20]. However, if the aim is to determine information of most importance, it is feasible that a large majority (for example greater than 70%) should rate the information “extremely important” [13]. Data from the first round of questionnaire surveys is summarised to produce feedback that may include data such as the mean group score. A second questionnaire containing the retained items is developed. It includes the individual participant responses from the first round and the mean scores of patients and professionals for each domain. Participants are then asked to re-prioritise each remaining domain in light of the feedback data provided.

A priori criteria are applied to the responses from the second Delphi round, and information domains considered highly important are retained. All other information domains are discarded from the process, and not included in the final CIS. These domains contain information that may still be discussed in consent consultations and prove important to any individual patient but are not, by definition, core information valued by a majority of stakeholders.

### Finalising the core information set

Information domains retained after the Delphi process can be discussed in group consensus meetings. These meetings aim to structure interaction within a group through a process of anonymous voting and subsequent discussion. Separate meetings for patients and professionals are recommended to enable patients to express their views freely. Consideration for inclusion in the CIS is debated through moderated discussion. Real-time voting technology (e.g. Turning Point©) can be used to collate stakeholders’ views on whether or not each domain should be included in the final set, whereby participants are asked to vote “in”, “out”, or “unsure” for each domain. Those domains clearly voted “out” by a majority are discarded. Those for which there is an equivocal result (“unsure” or an even split between “in” or “out”) are re-presented to the group. Discussion and re-voting continue until consensus is reached on every domain.

At the end of this phase, the CIS obtained from each consensus meeting will typically contain around 10 domains. An important final step is to discuss the content of the core information sets with consensus meeting participants in order to gauge its appropriateness and reflect on any dissenting opinion. If required, further voting and discussion can take place. The patient and professional sets are compared and then condensed into a single set of core information for informed consent to the specific procedure. Figure 1 uses a CIS developed for patients about to undergo oesophagectomy to summarise the suggested steps, and to illustrate the organization and prioritisation of large amounts of information to the core of eight domains [13].

## Discussion and conclusions

Medical intervention requires a patient’s authorisation. This ethical, legal, and professional requirement is realised through a process of information provision and exchange aimed at reaching informed consent. Despite a wealth of theory, guidance, and legal precedent, this area of clinical practice remains challenging [21]. Fundamental to the process should be the communication of information that is: useful and understandable to the patient; allows him or her to weigh up the potential benefits and risks of the procedure; and results in a decision that best fits his or her goals and circumstances [22–24]. Knowing how much and what kind of information about a given procedure achieves these aims is difficult. Practice might not meet bioethical ideals, patients may lack information, and clinicians may be at risk of litigation. Prominent bioethicists and others have realised that efforts are required to provide understandable information of relevance to patients and professionals [11, 21]. Core information sets are part of a potential solution. Consisting of information of agreed importance about a given procedure, they would reduce variation in the practices of information provision about a given procedure, and be a starting point for the development of interventions to improve informed consent.

The fact that a landmark legal case in England recently reignited debate about how best to inform patients about a proposed intervention only serves to highlight the timely need for our work [6]. We are not the first to attempt to address informed consent from a frontline, service-delivery based perspective but little previous work has had an impact on practice. A recent Cochrane review of trials of interventions that aimed to improve consent showed that most were developed in a superficial way, with little theoretical basis for how information about the medical procedure was selected for inclusion [25]. The interventions were not piloted before evaluation in randomised trials; little training was given to those using the interventions; fidelity to the intervention protocols was not reported; and the standard care comparator was poorly described and monitored in most studies. The majority of studies were, in addition, small and addressed only the patients’ perspectives on informed consent. That is, no intervention aimed to change clinicians’ practice. There was limited follow-up, thus failing to capture potentially important longer-term outcomes including litigation or decisional regret. It appears, therefore, that much effort goes into multiple small scale studies, but these do not subsequently improve daily clinical practice.

There is a need for a theoretical basis for developing interventions that involve patients in the process, and for these to be piloted in practice. Training those who will be delivering the intervention is also required because simply providing patients and clinicians with a list of information domains for discussion is unlikely to satisfy modern legal, ethical, and professional standards. Indeed, such an approach risks a regressive, paternalistic practice that is counter to accepted bioethical principles. These issues require an in-depth investigation that does not concentrate solely on the disclosure of risk but aims to develop an innovative approach to consent that is embedded in a model of doctor-patient communication that addresses those issues of importance and additional value to the patient, including survival and quality-of-life [26]. Before this, a proper understanding of the nature of what information is most important to patients and others is required.

The approach presented in this paper aims to establish what information is rated as most important by patients and clinicians for discussion during the informed consent process. It is one way of starting to address the broader issues related to information, including ensuring understanding, facilitating deliberation, and permitting recall. Key strengths include the systematic application of health services research methods. This allows a reproducible and transparent way of prioritising complex information. Patients are involved in every stage, which is crucial if we are to understand how and why patients prioritise core information. As discussed earlier, it is not the intention that CIS should encourage a ‘tick-box’ approach to informed consent consultations whereby clinicians view the domains within it as a panacea. This would be an undesirable outcome because the intention of this model is to foster a patient-centred approach to informed consent. The key to addressing this will be future work that assesses how best to implement core information sets in routine practice, as well as which outcomes should be measured in evaluative work that assesses their effectiveness.

The core information set could form the basis of an intervention to train clinicians to discuss these key issues of importance to patients. The core information set might, for example, be operationalised as a consultation agenda that prompts surgeons to discuss the domains with all patients while also seeking to engage patients in a two-way dialogue. The intervention might involve an initial, facilitated training session using simulated clinical scenarios in which trained medical actors play the role of patients and guided by suggested competencies for informed, shared decision-making [27]. Different scenarios would be used to train, and subsequently test, the surgeons’ abilities to use the core information set to gain patients’ consent (or not) to proceed with surgery. Consultation observation and scoring instruments have been developed in the field of shared decision-making and it might be feasible to apply aspects of these instruments to the assessment of recorded consent consultations. In addition, conversation analysis techniques could be applied that would help explain why certain ways of communicating the core information set result in better consultations. Interviews with surgeons after consultations would provide insight into positive and negative aspects of the intervention. The aim would be to gather evidence about what works, what does not work, and why? These initial stages, or pilot work, would serve as a field-testing of the core information set and examples of good practice would be developed into tools that help facilitate better consultations. We acknowledge that the model described above requires significant research and time resources. Future work will, in addition to addressing how to implement core information sets in practice, aim to streamline the process of development. There is scope to refine, and speed-up the initial steps in core information set development including addressing whether in-depth systematic reviews are required, and whether focus group work might work as well as individual, in-depth interviews. We also hypothesise that, based on core sets developed thus far, there will be areas of commonality and overlap between sets developed for different procedures (for example, ‘survival’ or ‘long term quality of life’ in surgical oncology). A growing repository of core information sets will help identify such areas of overlap in order to negate the requirement for some of the developmental work.

Our model is framed as a means of respecting an individual’s need for information in order to provide an autonomous authorisation, or refusal, of a proposed medical intervention. This justification for informed consent is not universally accepted, in part because of the fact that, in the majority of cases, treatment options are presented to patients by doctors and thus ‘true’ autonomy (where, for example, the patient presents with a request for a particular treatment and the expectation is that it will be carried out) does not result [9, 28]. It should be recognised that patients may not be equipped with a background level of training, knowledge, or understanding that would allow them to formulate treatment plans independently. It is on this latter construct of autonomy that our core information model is based. The aim of our work is to better equip patients with the information that helps them make the decision that is best for them within the resources available and to better equip doctors to guide patients through that process.

The proof of effectiveness of this model will require it to successfully deliver the outcomes of informed consent, however those are defined. At the very least, it will need to result in patients having sufficient understanding of the proposed treatment and its alternatives to make their authorisation or refusal of it meaningful to the *individual*. Linked to this is that the model must be viewed by clinicians as a credible tool that enhances consultations rather than hindering them, making them longer, or otherwise disruptive [29]. A key assumption made in this account of our model is that all patients have the capacity to fully participate in the informed consent process. Further work will be required to explore the applicability of this model in situations where capacity may be diminished. Similarly, the acceptability of core information to different ethnic groups, for whom information provision in the medical setting might be valued in different ways, will require careful and sensitive consideration.

To date, we have successfully applied these methods to define a core information set in one area of surgical oncology. Our early work suggests this approach is acceptable to patients and clinicians. Work is ongoing to develop other core information sets and we have begun the process of exploring how best to incorporate core information as an effective tool in clinical consultations which results in patients making decisions that reduce the likelihood of cases like *Montgomery* reaching the courts.

## References

[1] Sokol DK (2015). Update on the UK law on consent. BMJ.

[2] Bolam v. Friern HMC [1957] 2 All ER 118.

[3] Sidaway v. Bethlem Royal Hospital Governors [1985] AC 871

[4] Pearce v. United Bristol Healthcare NHS Trust [1999] 48 BMLR 118.

[5] Chester v. Afshar [2004] UKHL 41

[6] Montgomery v. Lanarkshire Health Board (Scotland) [2015] UKSC 11

[7] Heywood R (2015). R.I.P. Sidaway: Patient-oriented disclosure - a standard worth waiting for? Montgomery v Lanarkshire Health Board [2015] UKSC 11. Med Law Rev.

[8] Dire C (2015). Doctors must not cherry pick information to give patients, landmark case determines. BMJ.

[9] O’Neill O (2003). Some limits of informed consent. J Med Ethics.

[10] Edwards A, Elwyn G (2009). Shared decision-making in health care: Achieving evidence-based patient choice.

[11] Faden RR, Beauchamp TL (1986). A history and theory of informed consent.

[12] Elwyn G, Montori VM, Elwyn G, Edwards A, Thompson R (2016). Tools to engage patients in clinical encounters. Shared decision making in health care: achieving evidence-based patient choice.

[13] Blazeby JM, Macefield R, Blencowe NS, Jacobs M, McNair AG, Sprangers M (2015). Core information set for oesophageal cancer surgery. Br J Surg.

[14] McCartney M (2013). Patient information leaflets: “a stupid system”. BMJ.

[15] Blencowe NS, Strong S, McNair AG, Howes N, Elliot J, Avery KN, Blazeby JM (2015). Assessing the quality of written information provision for surgical procedures: a case study in oesophagectomy. BMJ Open.

[16] Hammersley M, Atkinson P (2007). Ethnography. Principles in practice.

[17] Williamson PR, Altman DG, Blazeby JM, Clarke M, Devane D, Gargon E, Tugwell P (2012). Developing core outcome sets for clinical trials: issues to consider. Trials.

[18] Charters E (2003). The use of think-aloud methods in qualitative research. An introduction to think-aloud methods. Brock Educ.

[19] Jones J, Hunter D (1995). Consensus methods for medical and health services research. BMJ.

[20] Hasson F, Keeney S, McKenna H (2000). Research guidelines for the Delphi survey. J Adv Nurs.

[21] Grady C (2015). Enduring and emerging challenges of informed consent. N Engl J Med.

[22] Department of Health. Reference guide to consent for examination or treatment. Available online at https://www.gov.uk/government/publications/reference-guide-to-consent-for-examination-or-treatment-second-edition. Accessed 20 Apr 2017.

[23] Royal College of Surgeons. Professional standards for cosmetic practice. Available online at https://www.rcseng.ac.uk/standards-and-research/standards-and-guidance/service-standards/cosmetic-surgery/professional-standards-for-cosmetic-surgery/. Accessed 20 Apr 2017.

[24] General Medical Council. Press Release. Give patients time to think before cosmetic procedures, doctors told. Available online at http://www.gmc-uk.org/news/26550.asp. Accessed 20 Apr 2017.

[25] Kinnersley P, Phillips K, Savage K, Kelly MJ, Farrell E, Morgan B (2013). Interventions to promote informed consent for patients undergoing surgical and other invasive healthcare procedures. Cochrane Database Syst Rev.

[26] Main B, McNair A, Davis L, Blazeby JM. Bringing informed consent back to patients. Available online at http://blogs.bmj.com/bmj/2014/08/05/barry-main-et-al-bringing-informed-consent-back-to-patients/. Accessed 20 Apr 2017.

[27] Towle A, Godolphin W (1999). Framework for teaching and learning informed shared decision making. BMJ.

[28] Huxtable R (2014). Autonomy, best interests and the public interest: treatment, non-treatment and the values of medical law. Med Law Rev.

[29] Wear S (1998). Informed consent. Patient autonomy and clinician beneficence within health care.

